# The cholera emergency is avoidable

**Published:** 2023-05

**22 March 2023 -** The world is facing an upsurge in cholera, even touching countries that have not had the disease in decades. Years of progress against this age-old disease have disappeared. While the situation is unprecedented, the lesson to draw is not a new one: safe drinking water, sanitation and hygiene are the only long-term and sustainable solutions to ending this cholera emergency and preventing future ones.

The global cholera situation is concerning, but as we mark World Water Day today, and the historic United Nations Water Conference begins in New York, the Global Task Force for Cholera Control (GTFCC) is appealing to countries and the international community to channel that concern towards concrete action.

First, to prevent future outbreaks, countries need strong public health surveillance systems to quickly identify and confirm cholera cases, allowing for immediate action. Countries with ongoing widespread outbreaks need immediate support to track and tackle the current crisis. We can’t solve a problem we can’t see.

Second, stop the cycle that takes us from emergency to emergency by investing in water, sanitation, and hygiene (WASH). When there is a cholera outbreak, responders rush in soap and chlorine tablets, bring in safe water in trucks, and build temporary latrines to prevent the outbreak from spreading. While these actions are undoubtedly life-saving, longer-term investments in WASH infrastructure can prevent outbreaks in the first place. Wherever in the world cholera has been eliminated, it has been thanks to improvements in basic water, sanitation and hygiene – access to these is an internationally recognized human right.

Moving from an emergency response to long-term improvements is more effective, because although cholera is a health issue, first and foremost, it is a development issue.

Third, focus efforts on cholera hotspots. Fighting cholera requires a targeted approach centred on hotspots–health zones or districts–where cholera cases are concentrated. Focusing on cholera hotpots more than doubles the return on investments in safe water, sanitation and hygiene: from US$4.30 to US$10 for every US$1 invested.

Fourth, support the development and implementation of national cholera plans, including the budget allocated for WASH. These national plans lay out multisectoral actions needed for sustained cholera prevention and control, including the use of oral cholera vaccines, putting communities at the center.

Poverty, conflict, and disasters continue to fuel cholera, now turbo-charged by climate change. The future presents multiple challenges, but at least for cholera, we have the answer: access to safe water, sanitation and hygiene in cholera hotspots. Urgent, targeted investments will get us there.

Available from: https://www.who.int/news/item/22-03-2023-the-cholera-emergency-is-avoidable

